# 40th Anniversary Virtual Issues: Human Genetic Diversification

**DOI:** 10.1093/gbe/evae033

**Published:** 2024-03-13

**Authors:** Casey McGrath

To celebrate the 40th anniversary of the first issue of *Molecular Biology and Evolution*, the Society for Molecular Biology and Evolution (SMBE) journals are launching a series of virtual issues in 2024. These virtual issues contain selected papers published in the society's journals, *Molecular Biology and Evolution* and *Genome Biology and Evolution*. Each virtual issue will be accompanied by a Perspective that highlights the historic and contemporary contributions of our journals to a specific topic in molecular evolution.

March's Perspective, titled “Biogeographic Perspectives on Our Species’ Genetic Diversification,” appears in *Molecular Biology and Evolution* and was written by Tábita Hünemeier. The accompanying virtual issues—1 for *Molecular Biology and Evolution* and 1 for *Genome Biology and Evolution*—include noteworthy papers on Human Genetic Diversification published in the 2 journals and can be found at SMBE's 40th anniversary site.

